# Cortisol and heart rate response of mares during the in‐hand breeding procedure with and without restraint

**DOI:** 10.1111/evj.70083

**Published:** 2025-09-02

**Authors:** Maria Fernanda Atayde, Beatriz Vidondo, Rupert Bruckmaier, Sabrina Briefer Freymond, Harald Sieme, Rebekka Rey‐Kaeser, Dominik Burger

**Affiliations:** ^1^ Swiss Institute of Equine Medicine Vetsuisse Faculty University of Bern Avenches Switzerland; ^2^ Veterinary Public Health Institute University of Bern Liebefeld Switzerland; ^3^ Veterinary Physiology, Vetsuisse Faculty University of Bern Bern Switzerland; ^4^ Agroscope Swiss National Stud Farm, Les Longs‐Prés Avenches Switzerland; ^5^ Clinic for Horses – Unit for Reproductive Medicine University of Veterinary Medicine Hannover Hannover Germany

**Keywords:** horse, in‐hand breeding, restraint methods, stress

## Abstract

**Background:**

In‐hand breeding involving restraint methods is likely the most common practice in the horse breeding industry worldwide. However, welfare issues that arise from such management have not been investigated in detail.

**Objectives:**

(1) To investigate whether the mares show increased stress responses during in‐hand breeding using (a) a lip twitch and (b) hobbles. (2) To evaluate if the use of restraint methods influences the mare's expression of oestrous behaviour.

**Study Design:**

Intra‐individual cross‐over design.

**Methods:**

Six Franches‐Montagnes stallions and 10 warmblood mares were used in these experiments. Each mare underwent standardised teasing and breeding sessions with and without one of the two restraint methods (morning and afternoon) on the day before ovulation. Salivary cortisol concentrations, continuous heart rate (HR) and heart rate variability (HRV) before, during and after the teasing and breeding sessions were measured as objective stress indicators. An ethogram was established to evaluate the behaviour during breeding.

**Results:**

Using the lip twitch increased cortisol concentrations *(*with twitch from 0.77 ± 0.07 to 1.20 ± 0.07 ng/mL vs. without twitch from 0.73 ± 0.07 ng/mL to 0.99 ± 0.07 ng/mL; *p = 0.04*), and the hobbles could not be applied to 2 out of the 10 mares in our study, as they did not tolerate this restraint method.

**Main Limitations:**

The sample contained a small number of mares.

**Conclusions:**

Using a lip twitch in mares during in‐hand breeding is accompanied by a slight degree of stress, while hobbles are tolerated very well or not at all by the mares.

## 1 INTRODUCTION

In‐hand breeding is likely the most commonly performed horse breeding practice across the world, for example, the entire Thoroughbred industry. It usually involves some restraint of the mare while a handler leads the breeding stallion to the mare and lets him mount the mare and breed her. In most cases, the precopulatory contact between the mare and the stallion is limited to a brief ‘teasing’ phase to check the mare's receptive behaviour. Commonly used practices for restraint of the mare are the application of a lip twitch (a device that applies pressure around the upper lip of the horse) or hobbles (tying the hind legs of the mare with ropes to a belt around her neck). These restraint methods are designed to prevent the mare from moving and/or kicking and therefore should minimise the risk of injuries for the stallion and the staff.^1^, ^2^

Nowadays, there are numerous questions regarding these practices and, in particular, how much they differ from natural mating observed in feral herds and therefore how much they prevent the fulfilment of behavioural needs.^3^, ^4^ In addition, the scientific basis of welfare issues, potential frustration and stress that arise from the management of breeding horses and the use of different restraint methods is almost entirely lacking.^5^ To assess the acute stress response in horses, different physiological markers have been documented. Measurements of heart rate (HR) and heart rate variability (HRV) represent validated methods to measure stress in horses.^6^, ^7^ Stressful situations will usually cause a reduction of variability between consecutive heartbeats (RR intervals) and an increase in mean HR, reflecting the changes in the balance between vagal (parasympathetic) and sympathetic stimulation. In addition, measurements of cortisol concentrations, in both serum and saliva, are used in human and animal studies as an indirect measure of stress,^8^ and salivary cortisol concentrations have been successfully used as an indicator of stress in equine studies investigating reproduction,^9^ transportation,^10^ training^11^ and competition.^12^, ^13^, ^14^, ^15^ Typical precopulatory interactions (i.e., teasing) observed in wild horses, and often absent in in‐handbreeding, are defined by the mare displaying a series of characteristic behaviours which inform the stallion of her willingness to breed.^3^ Ethograms describing the natural breeding in feral horses are well‐established,^3^ but none exist specifically for in‐hand breeding.

This study aimed to investigate whether the use of a lip twitch or hobbles could represent a stress factor during in‐hand breeding and also whether it influences the mare's expression of oestrous behaviour. It was hypothesised that restrained mares would show increased stress responses and less pronounced oestrous behaviour during in‐hand breeding compared to mares in which these restraint methods were not used.

## 2 MATERIALS AND METHODS

### 2.1 Infrastructures

**FIGURE 1:**
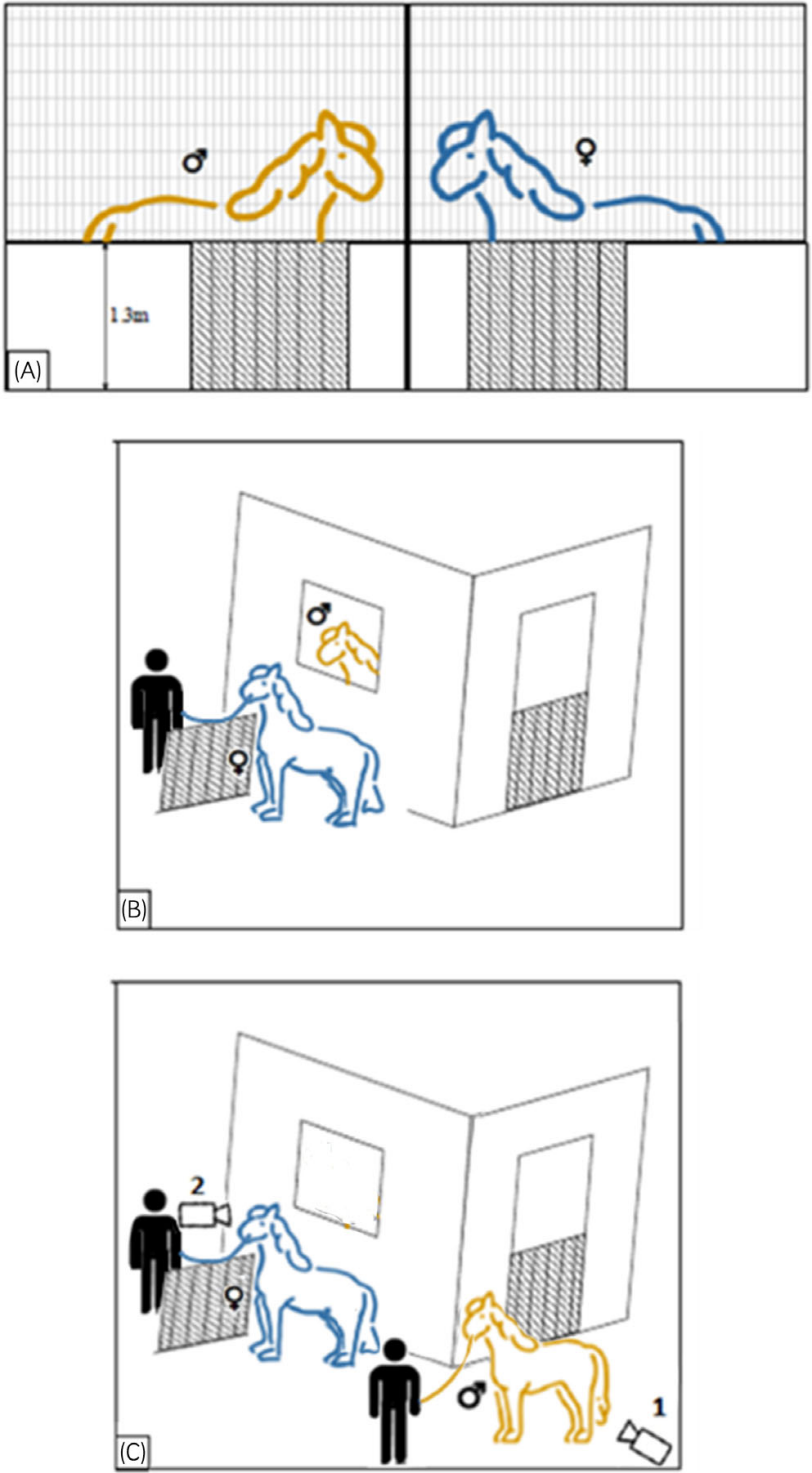
Habituation, teasing and breeding area. The stallion is illustrated in yellow and the mare in blue. (A) Habituation in box. (B) Teasing outdoors in a special area where the stallion was allowed to put his head out of the box. (C) Breeding area (same as the teasing area). Position of 2 cameras filming (1) the mare and the stallion and (2) the mare's head and neck (adapted from Szijarto^18^).

The study took place from March until August 2023 at the facilities of the ISME Avenches. The experimental infrastructures included a stable with two adjacent boxes for the stallion and the mare, separated by a wood separation (1.3 m height) and a squared metal grid (5 × 5 cm) (Figure 1A). The stallion box had an outside window towards the teasing and in‐hand breeding area. This consisted of a wooden wall on a non‐slip rubber floor (Figure 1B,C).

### 2.2 Animals

Six breeding stallions (Franches‐Montagnes horses, mean ± SD age = 17.3 ± 2.4 years) and 10 mares (Warmblood horses, without foal, mean ± SD age = 12.7 ± 1.7 years) were used for the experiments. All animals were used to being handled by humans. The mares were kept in a group paddock with stables bedded with straw and the stallions in individual boxes. The animals did not have contact between sexes other than during the experimental exposure.

Before the study, all mares experienced a minimum of one in‐hand breeding using a twitch as a restraint method.

### 2.3 Study design and procedures

Oestrus was induced in all mares with 37.5 μg of cloprostenol (synthetic analogue of prostaglandin F_2α_, Genestran, Graeub AG); the mares underwent standardised gynaecological examinations (ultrasonography per rectum of ovaries and uterus) and teasing (each mare 3 min/day with a stallion that was not involved in the study) until a follicle had reached a size of 35 mm or more, and uterus oedema was at stage >2^16^ combined with typical oestrous behaviours of the mare that include ‘sawhorse’ posturing with lowered pelvis, vocalisations, frequent urination and rhythmic eversion of the clitoris.

An intramuscular treatment of 1.25 mg deslorelin (BioRelease® Deslorelin, Caledonian Holdings Ltd) was then administered at 5:00 PM, before all experimental exposures on the following day, to induce ovulation ~36–42 h later.^17^ At 8:00 AM on the day after ovulation induction, the mare and the stallion were stalled in boxes separated by a wooden board and metal grid allowing visual and olfactory contact (Figure 1A: habituation phase of 10 min). After 10 min, the mare was moved from the box to the teasing area where she had the possibility for visual and olfactory contact, but also physical contact with the stallion through the window of the box. The mare was then led to the stallion to induce nose‐to‐nose contact (Figure 1B: teasing phase of 5 min). The person holding the mare (always the same in all phases) tried to interfere as little as possible, allowing the mare to express her oestrous behaviour.

The teasing phase was directly followed by in‐hand breeding with the same stallion that was used during the habituation and teasing phases (Figure 1C). To do so, the stallion was taken out of the box and brought to the mare. Eight hours later, at 4.00 PM, the procedure including the 10 min of habituation, 5 min of teasing and in‐hand breeding was repeated using the same stallion as in the morning.

**FIGURE 2:**
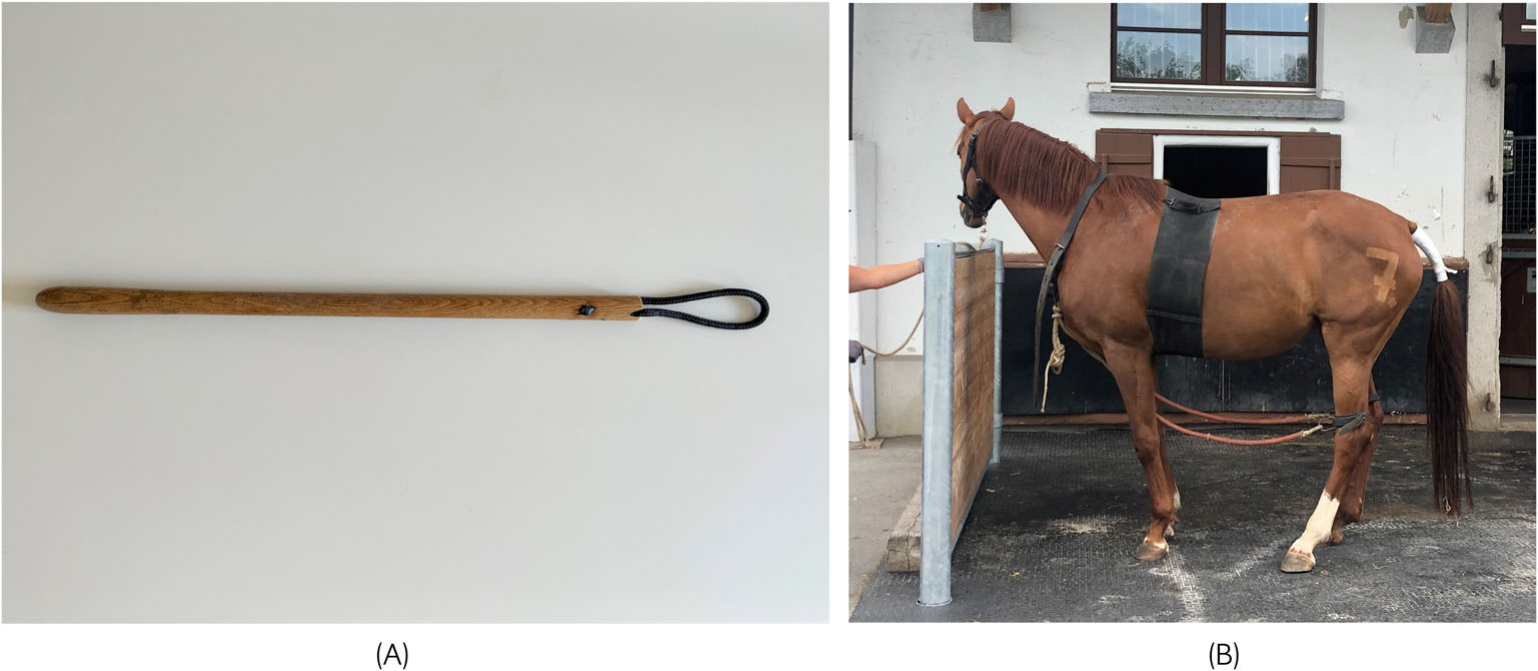
(A) Lip twitch used in the Experiment A; (B) Hobbles used in the Experiment B.

In Experiment A, half of the mares (random allocation) in the morning were prepared with a lip twitch (Figure 2A; length of the wooden handle: 73 cm, length of the rope loop: 17 cm) after the teasing phase, immediately before the in‐hand breeding. The other half of the mares did not undergo any restraint method for the morning in‐hand breeding. For the second in‐hand breeding in the afternoon, the restraint conditions were crossed; that is, the mares that had the lip twitch in the morning were not restrained in the afternoon, whereas the other 50% of the mares were prepared with the lip twitch immediately before the second in‐hand breeding in the afternoon. According to the within‐subject cross‐over design, the whole procedure was repeated during a second oestrous cycle in the reverse order.

In Experiment B, we proceeded precisely as in Experiment A except that this time we replaced the lip twitch with the application of the hobbles (Figure 2B) just before in‐hand breeding. Again, according to the within‐subject cross‐over design, this procedure was repeated during the following oestrous cycle in the inverse sequence of the procedures.

Following the teasing and breeding manipulations and continuing a standardised gynaecological protocol, mares were examined to determine the ovulation time. Eight days after ovulation, mares were again treated with cloprostenol to induce the next oestrous cycle.

### 2.4 Measurements

The data for heart rate (HR) and heart rate variability (HRV: Root Mean Square of Successive Differences (RMSSD), Standard Deviation 1 (SD1), Low frequency to high Frequency Ratio (LF/HF) and Standard Deviation of Normal‐to‐Normal Intervals (SDNN)) were continuously recorded with the non‐invasive monitoring system Polar Vantage V2. The Polar belt was fitted to the mares around the girth area, with the Polar transmitter placed on the left thorax, as described previously.^18^

We divided the time of the experiment into different time points: 1—baseline, 2—habituation phase inside the box, 3—teasing phase, 4—time period when the respective restraint device was installed, 5—time period from the moment the stallion came out of the box until he mounted the mare, 6—duration of copulation, 7—time period when the mare had been returned to the stable with the other mares. After the experimental period, obtained data was evaluated using Kubios HRV Software (Biomedical Signal Analysis Group, Department of Applied Physics, University of Kuopio, Finland).^19^

Saliva samples for cortisol analysis were collected five times from the mares using a cotton‐based swab (Salivette; Sarstedt). The salivette was held with a surgical artery clamp and inserted at the angle of the lips into the horse's mouth for at least 40 seconds until the cotton was well soaked with saliva. Samples were taken 5 min before the habituation phase, immediately after in‐hand breeding and 5, 20 and 60 min after in‐hand breeding, and were directly centrifuged at 185*g* for 10 min at room temperature and stored at −20°C for later analysis. To determine salivary cortisol concentrations, a competitive enzyme immunoassay (cELISA, Salimetrics, Newmarket, UK) was used with a sensitivity of <0.03 ng/mL according to the manufacturer. The assay has been previously validated for use in horses,^20^ determining intra‐assay and inter‐assay coefficients of variations of 6.4% and 4.0%, respectively.

To analyse the mares' behaviour, the teasing and breeding sequences were recorded using two different cameras (Sony® FDR‐AX33, Sony® HDR‐CX115E). One camera filmed the mares' head and neck only, and the other camera was placed in a position where both animals could be observed during the whole teasing and breeding time (see Figure 1C). In addition, the cameras recorded vocalisations during each procedure. The videos from the moment that the teasing phase finished until the stallion stepped down from the mare's back were analysed afterwards using a recently established modified ethogram adapted from previously published literature in feral horses (see Table 1) and the software ‘Observer’(Observer XT version 15, Noldus). Ten percent of the videos were selected randomly and watched twice by the same person to calculate the intra‐observer reliability, and 10% of the videos selected randomly were watched a second time by another observer to calculate the inter‐observer reliability. Intraclass correlation coefficient (ICC) was calculated using a two‐way mixed design to assess the absolute agreement between the scores of the two observers,^21^ with 0 indicating no agreement and 1 indicating full agreement. Generally, ICCs of 0.40 are considered poor, between 0.40 and 0.59 fair, between 0.60 and 0.74 good and those between 0.75 and 1.00 excellent.^22^

**TABLE 1:** Modified teasing and breeding ethogram adapted from previously published literature in feral horses.

Horse zone	Behaviour	Description
Uro‐genital region	Defecate^23^	The mare drops manure
	Urinate^3^	Urination and/or urination jet
	Winking^3^	Rhythmic eversion of the vulva, exposing the clitoris and lighter‐coloured internal membranes
Movement	Walk away^24^	The mare walks away from the stallion
	Lean on the wall^a^	The mare pushes her shoulder or/and flank against the wall
	Look for stallion^3^	The mare searches for the stallion
	Escape^24^	The mare escapes from the breeding area
	Turns the back to the stallion^3^	The mare turns the back to the stallion, swinging the hips towards the head of the stallion
Legs	Rear‐up^25^	The mare lift both forelegs with forehead higher than hindquarters
	Foreleg flexed^3^	The mare flexes one foreleg on the side near the stallion
	Pawing^26^, ^27^	Repetitive movement of the foreleg scratching the ground
	Lift hind legs^24^	Lift one or two hind legs a few centimetres from the ground without kicking the stallion
	Kick^27^	One or both hind legs are lifted and extended caudally, either straight back or sometimes arcing laterally
Tail	Tuck tail^27^	As the stallion approaches from the back the mare presses her tail between her legs
	Raise tail^27^	The tail is lifted dorsally of the perineum for a few seconds or more
	Tail swishing^27^, ^28^	The mare swishes her tail side to side repetitively
Face/ head	Head toss^26^, ^27^	Repetitive and sudden movement of the head up and down
	Presence of eye white^28^	The sclera (eye white) is visible
	Eyelids partially closed^28^	Eyelids closed or half closed for 2–5 seconds with frequent blinking
	Wide nostrils^26^	The nostrils are open and wide
	Nose to nose contact^3^	The mare moves her nostrils close towards the male and holds them there for a few seconds
	Snapping^25^	The mare opens her mouth by moving her lower jaw up and down vertically in a chewing motion. The lips are stretched over the teeth and do not meet. The corners of the mouth are pulled back and rounded
	Trembling of lower lip^a^	The mare drops her lower lip and it trembles
	Stallion investigations^3^	The mare allows that the stallion scents, licks and nuzzle her
	Bite^a^	The mare bites the stallion
Ears	Ears rotated back, behind vertical or flat^28^	Ears rotated back behind vertical or flat (both or one only) for ≥5 s
	Ears not tense^a^	Ears both looking forward, sideways or each in a different direction
Voice	Whining^27^	Emitting a long, high‐pitched vocalisation
	Squealing^27^	Emitting a short, sharp, high‐pitched vocalisation

^a^ Personal observations.

### 2.5 Data analysis

Statistical analysis was carried out using the NCSS 2022 software package (NCSS Statistical Software). Descriptive statistics, histograms and normality tests were performed for continuous outcomes. Differences in salivary cortisol concentrations, HR and HRV indices between restraint methods and time points were evaluated using repeated measures ANOVA, taking into account repeated measures per mare. The mare identifier was included as a subject factor, while the restraint method (as a binary variable yes/no) and the time point were included as ‘within’ variables (due to the cross‐over design). The effect of the time of the day (morning and afternoon) was tested as non‐significant, so data was pooled together (a maximum total of 40 observations per time point and restraint method, 10 mares × 2 morning × 2 afternoon trials; and 7 time points; thus 140 possible observations per restraint method, and a grand total of 280 observations). For the analysis of behavioural variables, non‐parametric Kruskal–Wallis tests or logistic regression were used depending on the nature of the outcome (non‐normal continuous or binary, respectively, see Table 2). The level of significance was set at *p* < 0.05.

**TABLE 2:** Results of Kruskal–Wallis tests and logistic regression comparing both methods for ethological variables/outcomes observed in mares bred with/without lip twitch and with/without hobbles (only breeding sequence).

Horse zone	Behaviour	Outcome		Without twitch			With twitch			*p*‐value	Without hobbles			With hobbles			*p*‐value
				*N*	Mean	SD	*N*	Mean /OR	SD/SE		*N*	Mean	SD	*N*	Mean/OR	SD/SE	
Uro‐genital region	Defecate	Yes/no	Binary	14	—	—	18	—	—		15	0.13	0.35	14	—	—	
	Urinate	Yes/no	Binary	14	—	—	18	0.27	2.29	0.11	15	—	—	14	2.60	2.61	0.32
	Winking	% of time	Continuous	14	10.27	23.84	18	3.41	7.86	0.50	15	8.12	15.58	14	1.61	3.39	0.30
Movement	**Walks away**	Yes/no	Binary	14	—	—	18	0.06	3.19	**0.01**	15	—	—	14	0.46	2.61	0.42
	Lean on wall	Yes/no	Binary	14	—	—	18	—	—		15	—	—	14	—	—	
	**Look for stallion**	Yes/no	Binary	14	—	—	18	0.11	3.22	0.06	15	—	—	14	0.14	2.34	**0.02**
	Turns the back to the stallion	Yes/no	Binary	14	—	—	18	—	—		15	—	—	14	—	—	
	Escape	Yes/no	Binary	14	0.14	0.36	18	—	—		15	—	—	14	0.67	2.72	0.69
Legs	Rear‐up	Yes/no	Binary	14	0.43	0.51	18	—	—		15	0.13	0.35	14	—	—	
	Fore leg flexed	Yes/no	Binary	14	—	—	18	—	—		15	—	—	14	—	—	
	Pawing	Yes/no	Binary	14	—	—	18	—	—		15	—	—	14	—	—	
	Lift hind‐legs	Yes/no	Binary	14	—	—	18	—	—		15	—	—	14	0.07	0.27	0.30
	Kick	Yes/no	Binary	14	0.07	0.27	18	0.76	4.31	0.85	15	0.20	0.41	14	—	—	
Tail	Tuck tail	Yes/no	Binary	14	0.43	0.51	18	1.67	2.05	0.48	15	—	—	14	0.88	2.10	0.86
	Raise tail	Yes/no	Binary	14	0.36	0.50	18	0.69	2.16	0.63	15	—	—	14	0.49	2.14	0.34
	Tail Swishing	Yes/no	Binary	14	0.14	0.36	18	0.35	3.60	0.42	15	—	—	14	—	—	
Face/head	Head‐toss	Yes/no	Binary	14	—	—	18	—	—		15	—	—	14	—	—	
	Presence of eye white	% of time	Continuous	14	16.34	25.75	18	8.96	15.93	1.00	15	9.97	19.29	14	5.26	15.42	0.18
	Eye lids partially closed	% of time	Continuous	14	2.75	10.29	18	8.09	14.97	0.15	15	—	—	14	—	—	
	**Wide Nostrils**	% of time	Continuous	14	8.87	11.39	18	20.49	18.80	**0.04**	15	16.95	16.63	14	16.83	17.93	1.00
	Nose to nose contact	Yes/no	Binary	14	—	—	18	—	—		15	—	—	14	—	—	
	Snapping	% of time	Continuous	14	1.90	4.83	18	0.07	0.29	0.37	15	0.25	0.97	14	1.48	5.53	0.92
	Trembling of Lower lip	% of time	Continuous	14	—	—	18	1.69	4.90	0.20	15	—	—	14	—	—	
	Stallion investigations	Yes/no	Binary	14	—	—	18	—	—		15	—	—	14	—	—	
	Bite	Yes/no	Binary	14	—	—	18	—	—		15	—	—	14	—	—	
Ears	Ears rotated back, behind vertical or flat	% of time	Continuous	14	—	—	18	—	—		15	—	—	14	—	—	
	Ears not tense	% of time	Continuous	14	45.84	28.25	18	55.62	35.49	0.25	15	43.21	21.33	14	42.50	23.68	0.78
Voice	Whining	Yes/no	Binary	14	0.07	0.27	18	—	—		15	0.13	0.09	14	0.50	3.63	0.59
	Squealing	Yes/no	Binary	14	—	—	18	—	—		15	0.13	0.35	14	—	—	

*Note*: Means and Odds Ratio and their corresponding SD or SE. Significant differences at *p* < 0.05 are highlighted in bold print.

Abbreviation: *N*, number of trials (10 mares and a maximum of 4 trials per method, 2 in the morning and 2 in the afternoon).

Part of the experiments involving two mares had to be excluded due to the stallion's inability to achieve vaginal penetration during the first breeding attempt; therefore, the need to mount a 2nd or a 3rd time to achieve ejaculation (for mare number 1 this included all trials using the lip twitch, and with mare number 10 all trials using the hobbles, in total 4 trials). In two mares, the experiments with the hobbles had to be abandoned for welfare reasons, and no data could be collected due to the mares reacting in panic when the hobbles were applied (in total 8 trials). This generated a total of 68 valid trials (out of 80 total trials).

Group sample sizes of 40 rendered 80% power to reject the null hypothesis of zero effect size when the population effect size (Cohen's *d*) is 0.56 (medium) and the significance level (alpha) is 0.05. Additionally, we estimated that group sample sizes of 10 mares achieved 80% power to reject the null hypothesis of equal means when the population mean difference is *μ*1 − *μ*2 = 1.0–1.1 = −0.1 with a standard deviation for both groups of 0.1 (as observed for our salivary cortisol concentrations) and with a significance level (alpha) of 0.05 (PASS Power Analysis and Sample Size software, NCSS). The reduced sample size for matched pairs resulted in an achieved power of 0.75 (for the twitch experiments *n* = 9) or 0.68 (for the hobbles experiments *n* = 8).

## 3 RESULTS

### 3.1 Cortisol

**FIGURE 3:**
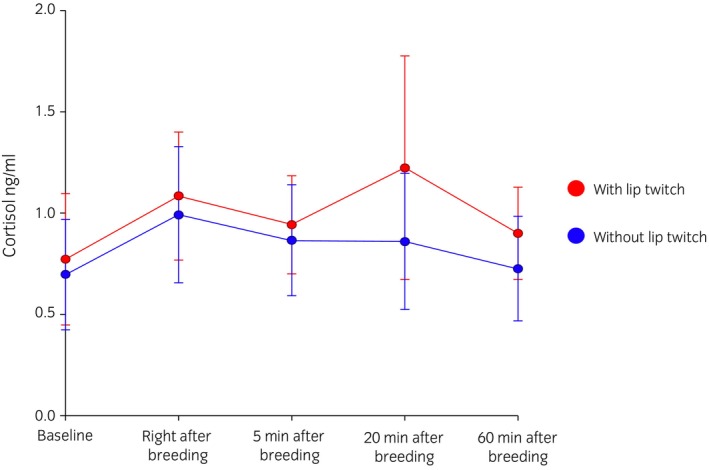
Scatter plot with standard deviation illustrating the evolution of mean salivary cortisol concentrations (in ng/mL) over time in 9 mares being restrained with a lip twitch or not and then bred.

**FIGURE 4:**
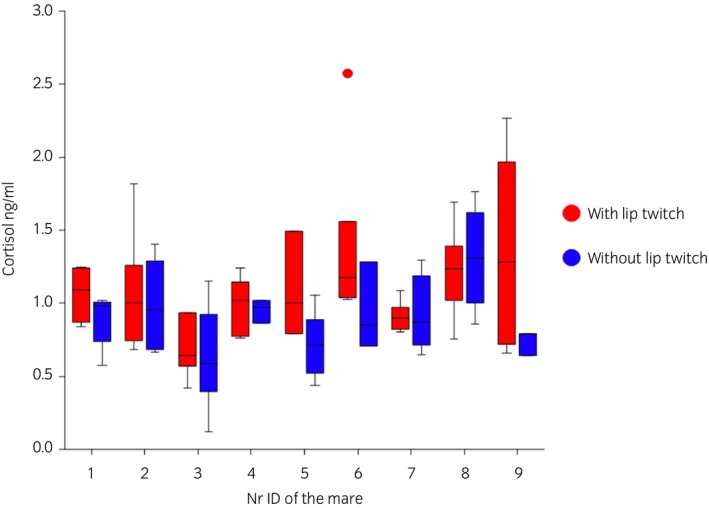
Differences of mean salivary cortisol concentrations (in ng/mL) in 9 mares being restrained with a lip twitch or not and then bred: Mean of all values right after breeding, 5 and 20 min after breeding for a given mare.

The time‐course of salivary cortisol concentrations in the experiments with or without the lip twitch are illustrated in Figure 3. With the twitch, immediately after mating, salivary cortisol concentrations increased from baseline 0.77 ± 0.07 ng/mL to 1.11 ± 0.07 ng/mL, reaching the highest values 20 min after breeding (1.20 ± 0.07 ng/mL). One hour after breeding, the cortisol concentrations had decreased to 0.90 ± 0.07 ng/mL. Without the twitch, there was an increase of cortisol concentrations from baseline (0.73 ± 0.07 ng/mL) to 0.99 ± 0.07 ng/mL right after breeding, which was the highest value over time. One hour after breeding, the cortisol concentrations had decreased to 0.74 ± 0.074 ng/mL. These changes in cortisol concentrations over time were significant (*p* < *0.01*). The differences between using the lip twitch and not using the lip twitch were also significant (*p* = *0.04*, Figure 4). In addition, an influence of the individual mare (*p* = *0.02*) on the effect of the twitch was evident, five mares showing a higher mean cortisol concentration during the experiment when using the lip twitch, one mare a slightly lower level and three mares no important changes.

**FIGURE 5:**
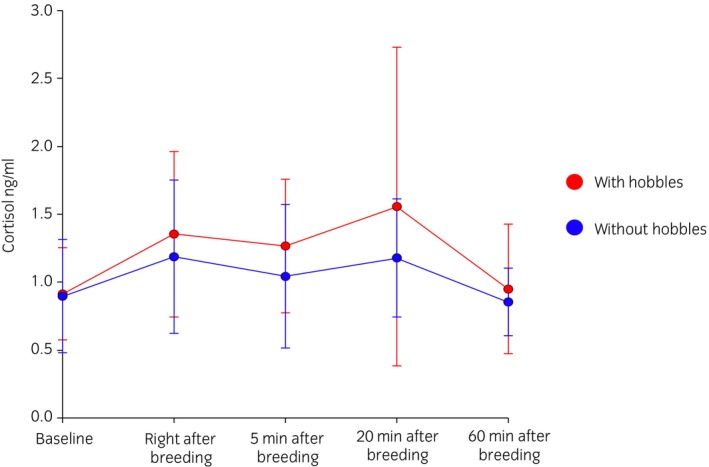
Scatter plot with standard deviation illustrating the evolution of mean salivary cortisol concentrations (in ng/mL) over time in 8 mares being restrained with hobbles or not and then bred.

Besides an influence of the individual mare on the effect of the hobbles (*p* = *0.01*), no evidence of differences over time (*p* = *0.22*) nor an effect of the restraint method (*p = 0.52*) was found when comparing the cortisol concentrations in the hobbles experiment (Figure 5).

### 3.2 Mean heart rate and heart rate variability

Mean HR increased at the moment the lip twitch was applied to 58.46 ± 2.46 bpm (baseline 39.81 ± 2.05 bpm). HR was highest as the stallion approached the mare and mounted (62.81 ± 2.23 bpm). During copulation, the HR decreased to 54.74 ± 2.17 bpm, and to 41.28 ± 2.11 bpm when the mares were back in their stable. Without the lip twitch, the mean HR after teasing also increased to 63.48 ± 2.23 bpm (baseline 39 ± 2.05 bpm), and the highest values were also measured when the stallion approached the mare and mounted (77.13 ± 2.23 bpm). These changes over time were significant (*p* < *0.001*) with a significant mare effect (*p* < *0.001*). However, there was no influence of the lip twitch on the mean heart rates (*p* = *0.99*). RMSSD, SD1, LF/HF and SDNN values demonstrated significant differences between mares (all *p* < *0.01*) and across time points (all *p* < *0.01*) while no effect of the lip twitch was found.

Regarding the hobbles experiments, the influence of the individual mare (*p* = *0.04*) and changes over time (*p* < *0.001*) on HR were significant, but the restraint method itself was not (*p* > *0.9*). When using the hobbles, immediately after being applied, the mean HR increased to 52.60 ± 1.77 bpm (baseline 42.30 ± 1.71 bpm). The highest values were found again during the period when the stallions approached the mare and mounted (60.40 ± 2.06 bpm), and they decreased during copulation (57.36 ± 1.77 bpm). Once the mares were back in their stable, the mean HR was 42.52 ± 1.77 bpm. Without the hobbles, the mean heart rates after teasing also increased to 57.02 ± 1.98 bpm (baseline 39.73 ± 1.71 bpm), and the highest values were also measured during the period when the stallions approached the mare and mounted (65.27 ± 1.83 bpm). Regarding RMSSD values, there was a mare effect (*p* < *0.001*) and there was also a significant difference across the different time points (*p* < *0.01*). Similarly, SD1 showed significant differences both between mares (*p* < *0.001*) and time segments (*p* = *0.02*). No evidence of differences was found for LF/HF values, while SDNN was significantly different between mares (*p* < *0.001*). No effect of the restraint method on RMSSD, SD1, LF/HF or SDNN values was detected.

### 3.3 Behavioural observations

Regarding the intra‐observer reliability, the comparison of the first and second scoring revealed high reliability for all outcome measures (ICC: range = 0.88–1). Concerning the inter‐observer reliability, the analysis revealed good to excellent agreement between the scores of the two observers for all outcome measures (ICC: range = 0.64–1). Only the behaviour ‘tuck tail’ was considered to be of fair agreement (ICC = 0.44).

The ethogram results are detailed in Table 2. Without the lip twitch applied, the mares walked away more often than when restrained (‘walks away’: *p* = *0.01*). With the lip twitch, mares more often showed ‘wide nostrils’ (20.49% vs. 8.87% without lip twitch; *p* = *0.04*). Some behaviours were shown with insufficient frequency to perform statistical analysis. Specifically, this applies to the ‘escape’ behaviour, which was the case in 2 out of 14 trials without the lip twitch, but never with the lip twitch, and it applies to ‘rear up’, which was observed in 6 out of 14 trials without the lip twitch versus never with the lip twitch. The behaviours ‘lean on wall’, ‘turns the back to the stallion’, ‘foreleg flexed’, ‘pawing’, ‘head‐toss’, ‘nose to nose contact’, ‘stallion investigations’, ‘bite’, ‘ears rotated back behind vertical or flat’ were never shown.

When assessing the potential effects of the hobbles, the only behaviour that differed was ‘looking for the stallion’ (*p* = *0.02*), which was expressed by 67% of the mares without hobbles applied versus 21% of those with hobbles. Some of the behaviours were also not observed in sufficient numbers for further statistics, for example, ‘rear up’ (seen in 2 out of 15 trials with no hobbles, but never with hobbles), ‘kick’ (3/15 trials without hobbles vs. 0/14 trials with hobbles) and ‘squealing’ (3/15 trials without hobbles vs. 0/14 trials with hobbles).

## 4 DISCUSSION

This study provides physiological data on mares during in‐hand breeding. Our results show that the application of the lip twitch significantly but individually increased salivary cortisol concentrations, peaking 20 min after breeding, while mares without the lip twitch showed a smaller increase. The cortisol response to the use of hobbles was not remarkable, with no evidence of an increase compared to the control condition. No consistent effect of the restraint methods applied was found on the mean HR or HRV indices.

Interestingly, individual variation among mares played a significant role in cortisol and HR outcomes and behavioural observations. This suggests that experience with restraint and in‐hand breeding, and intrinsic factors, such as the mares' temperament and physiological differences in stress reactivity, may influence their responses, as was also found in another recent study.^29^ The brief duration of the breeding phase, which lasted <1 min, and the procedure of the in‐hand breeding itself reduced the expression of natural behaviours commonly observed in nature, reducing the opportunity to see all the suggested behaviours found in the teasing phase and previously described in the literature.

Regarding the effect of lip‐twitching horses in veterinary procedures, one previous study^30^ showed that the lip twitch significantly decreased HR and increased HRV when applied for 5 min, suggesting a calming, probably analgesic effect. However, when the lip twitch was applied for a longer period, HR significantly increased and HRV decreased, indicating elevated stress. The short‐term decrease in HR and increase in HRV was attributed to a transient reduction in cortisol release, whereas the prolonged use likely leads to a stress‐induced rise in cortisol, further exacerbating the sympathetic response.^30^ In our study, as expected, HRV parameters aligned with the observed mean HR patterns, as seen in other studies: During the time points where mean HR increased, RMSSD, SDNN and SD1 values decreased, while LF/HF increased.^19^, ^29^ Despite these variations, we identified no significant differences in HR and HRV when using the lip twitch or not.

Regarding cortisol concentrations, the significant differences observed when applying the lip twitch demonstrate that although the HR and the HRV values did not show significant differences, a certain amount of stress is present when using this restraint method. The peak 20 minutes after the mating procedure can be attributed to this, as cortisol increases in saliva have been described in horses 20–30 minutes after the increase in blood concentrations.^8^, ^12^ However, the cortisol peak we observed was not as high as the increases that were found in other stress studies in adult horses.^10^, ^12^, ^14^ The mean salivary cortisol in our experiments using the lip twitch peaked at 1.20 ± 0.07 ng/mL, while studies involving transportation reported the highest cortisol concentrations of 3.10 ± 0.42 ng/mL.^10^ In different types of competition, for example, 5–7 min of dressage, values of 4.72 ± 4.28 ng/mL were measured, whereas after cross‐country and showjumping, values of 6.78 ± 6.54 ng/mL and 5.98 ± 4.89 ng/mL have been reported.^14^ Another study^12^ describing the salivary cortisol concentrations during a showjumping competition found values peaking at 1.92 ± 1.33 ng/mL 20 min after entering the competition. All these results indicate that the stress related to natural breeding with the short‐term use of a lip twitch found in our study, although significant compared with not using it, is minor compared to other activities.

Regarding the behaviours observed, the use of the lip twitch proved its efficient use as a restraint method, contributing to the observation that ‘walking away’ was seen more in the trials where the mare was not restricted. As for the ‘wide nostrils’ behaviour, this was more frequently observed in experiments involving the lip twitch and can be attributed to an elevated stress response caused by the physical and physiological effects of the lip twitch. Increased respiratory rates, which were reported in another recent study with the use of the lip twitch,^29^ likely led to the increased widening of the nostrils as the body attempts to meet oxygen demands.^31^

Regarding the behaviours during the hobbles trials, there was only a significant difference in the behaviour ‘looking for stallion’. Although hobbles appeared to have a milder physiological impact in most of the mares, it is important to acknowledge that they may still induce discomfort or distress in certain mares. This is evidenced by two mares in our study where the application of the hobbles led to panic responses, making it impossible to complete the experiments with the mares in question using this restraint method.

After the teasing phase, the short separation of the mare from the stallion resulted in a marked increase in HR, with a peak observed when the mare perceived the stallion's approach from behind. This physiological response may indicate that, despite prior interactions during the teasing phase, there are higher levels of stress due to the change in social environment and the way of approach made by the stallion. Indeed, observations in feral horses show no separation of the mare from the stallions at all, and most stallions do not approach the mare directly from behind. This finding emphasises the role of social interactions in equine reproductive behaviour and physiological responses.^31^, ^32^, ^33^ Notably, upon copulation, a decrease in HR was recorded, suggesting a potential calming effect. These observations are generally confirmed by the existing literature, which indicates that horses possess complex social structures and that modifications in their social environment can elicit significant physiological responses.^32^

## 5 LIMITATIONS

We used a small number of mares; however, substantial efforts were undertaken to create an appropriate study design and to standardise test conditions (e.g., controlling environmental factors, always using the same people to handle the horses and consistent protocols) and to use statistical techniques that consider multiple variables to ensure the results were thorough and reliable.

We applied the ‘best practice procedure’ carried out at many places, however, the study may not be representative of the mare population worldwide as, for example, not everywhere are only mares that show oestrous behaviour covered or is a 10‐min habituation between mare and stallion allowed. However, these two points were mandatory to obtain authorisation for our trials. In order not to interrupt the continuous interactions between the mare and the stallion, a cortisol analysis after the habituation phase and after teasing but before breeding was not carried out which makes it impossible to completely distinguish between the effect of applying a restraint method and the effect of breeding.

## 6 CONCLUSIONS

The present study revealed valuable information about mare physiology during in‐hand breeding, contributing to a deeper understanding of stress responses, physiological changes and ethical considerations in the context of recent criticisms of in‐hand breeding.

The findings indicate that short‐term restraint methods, such as the lip twitch, result in minor stress for the mares involved and may be appropriate for certain scenarios to enhance safety and the handling of both the mare and stallion by the people involved. This aligns with recent studies that suggest using minimal restraint can effectively mitigate risk to handlers and the stallions without severely impacting the mare's welfare.^29^

For those mares that accepted the use of the hobbles, no evidence of stress was detected. However, it may present an unpredictable risk in highly responsive horses, including Warmblood, Thoroughbred and Arabian horses, where sudden movements or situations could easily cause stress and sudden responses, compromising the safety of the people and animals involved. Given these concerns, the use of hobbles should first be tested to ensure that the mare will tolerate restraint by this method before the in‐hand breeding occurs to avoid accidents. In addition, integrated safety release systems are recommended, which enable rapid release of the hobbles in the event of high tension.

## FUNDING INFORMATION

Foundation Pro Pferd, Switzerland funded this project.

## CONFLICT OF INTEREST STATEMENT

The authors have declared no conflicting interests.

## AUTHOR CONTRIBUTIONS

**Maria Fernanda Atayde:** Methodology; investigation; formal analysis; visualization; writing – original draft. **Beatriz Vidondo:** Validation; formal analysis; data curation; writing – original draft. **Rupert Bruckmaier:** Investigation. **Sabrina Briefer Freymond:** Methodology; software; investigation. **Harald Sieme:** Conceptualization; methodology; writing – review and editing. **Rebekka Rey‐Kaeser:** Conceptualization; methodology; investigation; supervision; writing – original draft; writing – review and editing. **Dominik Burger:** Conceptualization; methodology; data curation; validation; formal analysis; supervision; funding acquisition; project administration; resources; writing – original draft; writing – review and editing.

## DATA INTEGRITY STATEMENT

Dominik Burger had full access to all the data in the study and takes responsibility for the integrity of the data and the accuracy of the data analysis.

## ETHICAL ANIMAL RESEARCH

The study was carried out and was approved by the Animal Health and Welfare Commission (Permission no. VD3860).

## INFORMED CONSENT

Written informed consent from the mare and stallion owners was obtained at enrolment into the study.

## Data Availability

The data that support the findings of this study are openly available in Zenodo at https://zenodo.org/records/16917283.
